# The Efficacy of Nitrates for Bone Health: A Systematic Review and Meta-Analysis of Observational and Randomized Controlled Studies

**DOI:** 10.3389/fendo.2022.833932

**Published:** 2022-02-10

**Authors:** Weibing Liu, Zhuoran Meng, Ge Wang

**Affiliations:** ^1^ Department of Orthopedics, The First People’s Hospital of Yunnan Province, The Affiliated Hospital of Kunming University of Science and Technology, The Key Laboratory of Digital Orthopedics of Yunnan Province, Kunming, China; ^2^ School of Basic Medical Sciences, Yunnan University of Chinese Medicine, Kunming, China

**Keywords:** nitrates, bone health, fracture, bone mineral density, meta-analysis

## Abstract

**Background:**

Although some studies have found that nitrates were beneficial for bone health, the findings are inconsistent. To assess the efficacy of nitrates for bone health, we conducted a meta-analysis.

**Methods:**

PubMed, EMBASE databases, Cochrane Library for relevant articles published before December 2021 were searched. All observational and randomized controlled studies that reporting bone mineral density (BMD), fractures with nitrates use were included. A meta-analysis was performed to calculate risk ratios (RRs) for fractures, change differences for bone mineral density.

**Results:**

Four cohort studies and two case-control studies examining the association between nitrates use and fractures were identified. The nitrates use was not associated with any fracture risk (RR = 0.97; 95% CI, 0.94–1.01; *I* ^2^ = 31.5%) and hip fracture (RR = 0.88; 95% CI, 0.76–1.02; *I* ^2^ = 74.5%). Subgroup analyses revealed no differences in fracture risk, whereas two cohort studies revealed a reduced risk of hip fracture (RR = 0.71, 95% CI, 0.58–0.86, *I* ^2^ = 0.0%). There were no statistically significant differences in BMD percent changes at lumbar spine (WMD = -0.07, 95% CI,-0.78–0.65; *I* ^2^ = 0.0%), total hip (WMD = -0.42, 95% CI,-0.88–0.04; *I* ^2^ = 0.0%), femoral neck (WMD = -0.38, 95% CI,-1.02–0.25; *I* ^2^ = 0.0%), or total body (WMD = -0.17, 95% CI,-0.51–0.17; *I* ^2^ = 0.0%) in two randomized controlled trials (RCTs) compared with a placebo. Another two RCTs compared nitrates with alendronate. Nitrates were comparable to alendronate in increasing bone mineral density at lumbar spine (WMD = 0.00, 95% CI,-0.01–0.02; *I* ^2^ = 0.0%). Besides, the most common adverse effect was headache, contributing to low adherence to therapy.

**Conclusion:**

Our meta-analysis showed no association between nitrates use and fractures in observational studies. The results of RCTs on the usage of nitrates and their effects on BMD were inconsistent. High-quality, long-term studies are needed to clarify the efficacy of nitrates for bone health.

## Introduction

Osteoporosis, defined as a decrease in bone mineral density (BMD) and an increase in bone fragility, is a major public health issue that affects both men and women around the world (1, 2). The population aged 50 or more who are at high risk of osteoporotic fracture was predicted to be 158 million in 2010, and this number is expected to double by 2040 (3). Bone fractures are connected with significant disability and morbidity, as well as a significant financial burden on injured individuals (4).

Nitrates (isosorbide mononitrate, isosorbide dinitrate, nitroglycerin), which are a type of angina medicine (5), appear to have beneficial effects on bone. These drugs, which act as nitric oxide donors, uncouple bone resorption and formation, resulting in improved bone metabolism (6). Nitric oxide has been shown to regulate osteoclasts, which are responsible for bone resorption (7). Besides, low NO levels have been shown to improve osteogenic proliferation, differentiation, and survival (8). However, higher concentrations inhibit osteoclast differentiation and survival (9). Animal studies have suggested that nitric oxide donors may increase bone mass by regulating osteoblast and osteoclast functions in ovariectomized mice (10). According to two epidemiological studies (11, 12), people who use nitrates had higher BMD and lower rates of bone turnover. However, one cohort study found no evidence that nitrate use was related to a decreased incidence of fractures or a higher BMD (13). Furthermore, the results of two randomized controlled trials (RCTs) that examined the effects of nitroglycerin ointment on BMD were contradictory (14–16).

Recently, several clinical trials evaluating the efficacy of nitrates for bone health have been reported. To our knowledge, no comprehensive meta-analysis on this topic has been performed. To determine the effect of nitrates on bone health, a comprehensive systematic review and meta-analysis based on an extensive search of observational and randomized controlled trials is required.

## Methods

### Search Strategy

The Preferred Reporting Items for Systematic Reviews and Meta-analyses guidelines were used for randomized controlled trials (RCTs) (17), and the Meta-Analyses and Systematic Reviews of Observational Studies guidelines were used for observational studies (18). Two independent reviewers (Liu and Wang) systematically searched PubMed, EMBASE database, Cochrane Library for relevant articles published before December 2021. An experienced librarian was consulted to generate a list of keywords and MeSH terms to conduct the search. The detailed search strategies are described in the **Supplementary Table 1**. Additional researches were discovered by searching the references of relevant research and review publications.

### Selection Criteria

Eligible studies were included if they fulfilled the following criteria (1): cohort studies, case-control studies, or randomized controlled trials (2), reported on bone mineral density (BMD), incident fractures with nitrates use (3), the reference group were non- nitrates users (3), studies provided adequate data for the efficacy estimates. The exclusion criteria were as follows (1): duplicate articles (2), molecular biology or animal research, and (3) reviews, case reports, letters, editorials, and meta-analyses. Two investigators (Liu and Wang) independently screened the articles by title and abstract after removing duplicate articles. Then, the full texts were obtained to identify the eligible studies. Disagreements in the study selection process were fully discussed and resolved through consultation with Meng.

### Data Extraction and Quality Assessment

The following information was extracted from each study: the first author’s name, the year of publication, the study design, the country, the interventions and co-interventions, the sample size, age, BMD, the duration of follow-up, and reported outcomes, including effect sizes (risk ratios (RRs), odds ratios (ORs), hazard ratios (HRs), BMD percent change, or BMD change) and adverse events. We extract the reported outcomes of the final time point for RCTs. If standard deviations were not reported, we used the confidence intervals to calculate the standard deviation. We used image extraction software (Engauge Digitizer) to extract data presented only in figures without corresponding numerical data.

We evaluated the quality of included RCTs using the Cochrane Risk of Bias tool (19), the quality of included observational studies was evaluated using the Newcastle–Ottawa Scale (NOS) (20). The data extraction and quality assessments were conducted independently by two authors (Liu and Wang).

### Data Analysis

The Stata 12.0 software was used to conduct the analysis. ORs were used as approximations of RRs since the incidence of fracture is so low (less than 5% per year). HRs, ORs, and RRs were extracted from the included studies. The pooled risk ratios (RRs) with 95% confidence intervals (CIs) from HRs and ORs were calculated using a random-effects model. Because most RCTs provided within-group changes in BMD outcomes, we used the reported or computed difference between the nitrates and reference groups as the effect size measure in the meta-analysis for BMD outcomes. We conducted meta-analyses when data from at least two trials were sufficiently homogenous in terms. To measure heterogeneity across trials, the *I* ^2^ and *Q* statistics were used. *I* ^2^ > 50% and *P* < 0.05 showed high heterogeneity across the studies examined. When significant heterogeneity was detected, subgroup analyses were performed to investigate the reasons for the heterogeneity. The Begger and Egger test was used to assess the publication bias of the studies included in the final analysis.

## Results

**Figure 1:**
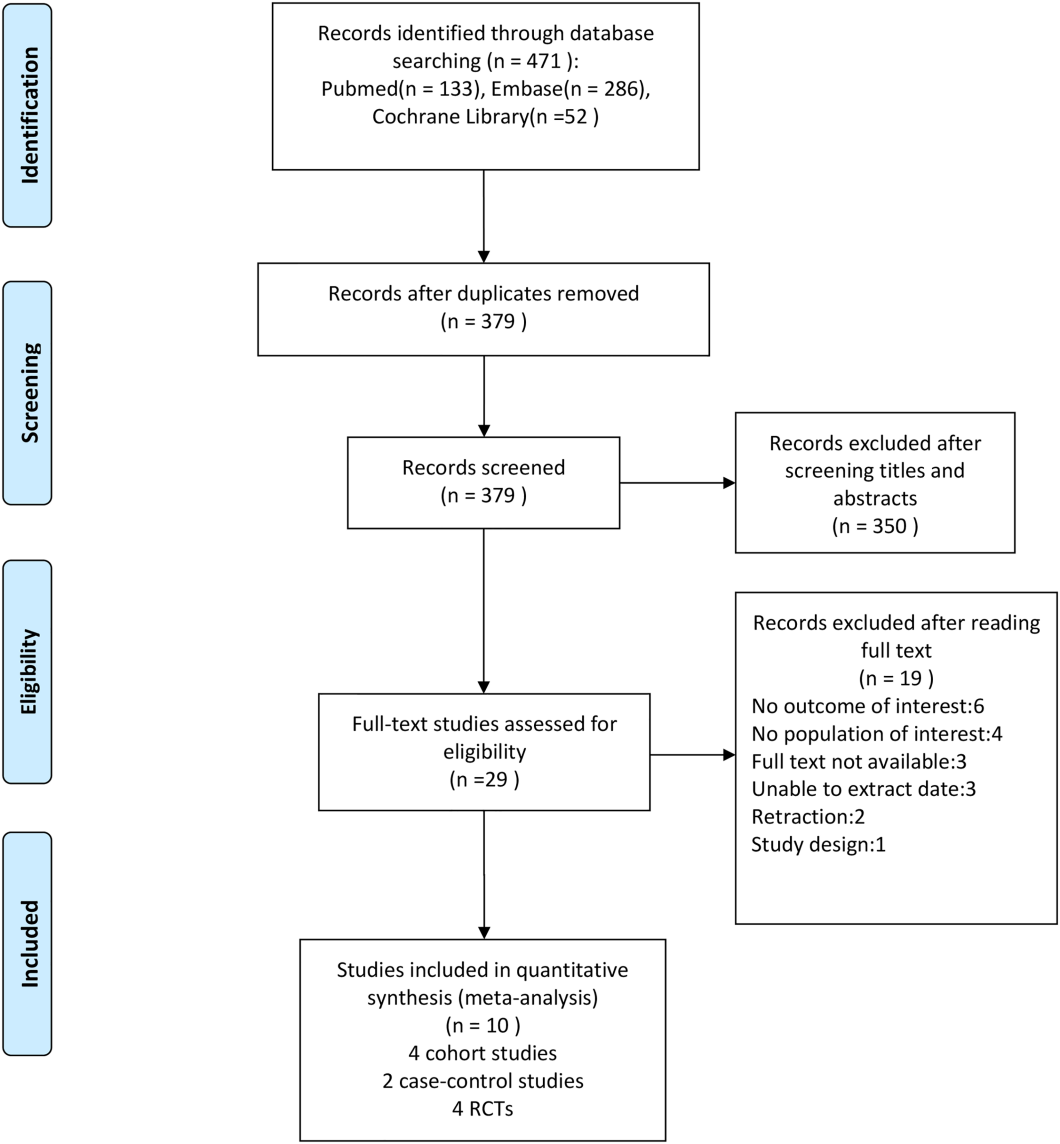
Flow diagram of the literature search process and study inclusion.

After conducting a literature search, we discovered 471 possibly eligible studies. After removing duplicates from the 471 papers retrieved, 379 were left, with 29 of them being chosen as potentially suitable after reviewing the titles and abstracts. After examining full texts, 10 were included for data extraction in our meta-analysis (four cohort studies, two case-control studies, and four RCTs). The literature search process is illustrated in **Figure 1**.

### Study Characteristics

There were 10 (11, 13–16, 21–25) studies included in our meta-analysis. Detailed characteristics of the included studies are presented in **Tables 1** and **2**. They were published between 1998 and 2020, including four cohort studies, two case-control studies, and four randomized controlled trials. Three studies were conducted in North America, four in Europe, one in Oceania, one in Asia, and one in Africa. Six studies reported BMD, and six studies reported fractures. Besides, two studies compared nitrates with a placebo, and two studies compared nitrates with alendronate. As indicated in **Table 1**, the NOS scores ranged from eight to nine points, indicating that all the observational studies chosen were of good quality. We classified RCT studies as having a low, uncertain, or high risk of bias (**Table 3**). There are two studies with a low risk of bias, one study with an uncertain risk of bias, and one study with a high risk of bias.

**Table 1:** Characteristics of the six included observational studies.

Author (year)	Study design	Country	Study population characteristics	Sample size (treatments or cases/controls)	Nitrate types	Mean age (Year) (treatments or cases/controls)		Study period	Outcomes	NOS quality score
Jamal et al. (11)	Cohort	USA	Elderly women	Daily(n=317), Intermittent(n=74)/Nonusers(n=5827)	NG, ISDN or ISMN	79 ± 5; 77 ± 5	76 ± 5	1992-1994	BMD, Fracture risk (HR)	8
Torstensson et al. (24)	Cohort	Denmark	Aged 65 years or older	66931/725692	Nitrates	70.6 ± 8	77.3 ± 7.5	1999-2012	Fracture risk (HR)	9
Golchin et al. (13)	Cohort	USA	Postmenopausal women	137564/1647	NG, ISDN or ISMN	63.1	67.9	1993-1998	BMD, Fracture risk (HR)	8
Misra et al. (23)	Cohort	UK	60 years or older with diagnosis of ischemic heart disease	14451/14451	NG, ISDN or ISMN	72.4 ± 7.6	72.4 ± 7.6	1986-2011	Fracture risk (HR)	8
Rejnmark et al. (22)	Case-control	Denmark	Danish population	124655/373962	NG, ISDN or ISMN	42	42	1977-2000	Fracture risk (OR)	9
Pouwels et al. (21)	Case-control	Dutch	At least 18 years old	6763/26341	NG, ISDN or ISMN	>18	>18	1991-2002	Fracture risk (OR)	9

NG, nitroglycerin; ISDN, isosorbide dinitrate; ISMN, isosorbide mononitrate.

**Table 2:** Characteristics of the four included randomized controlled studies.

Author (year)	Study design	Country	Intervention	Sample size (T/C)	Mean age (year) (T/C)		Mean BMD (g/cm^2^) (T/C)		Duration	Reported outcomes	Risk of bias
Wimalawansa et al. (15)	RCT	USA	NG (22.5mg daily) vs placebo	93/93	56.5 ± 4.2	55.3 ± 4.2	1.1 ± 0.1	1.1 ± 0.1	36 months	BMD percent change, body bone mineral content, height, adverse event	Unclear risk
Bolland et al. (14)	RCT	New Zealand	ISMO(20mg daily), ISMN(30mg/60mg daily) NG(25mg/50mg daily) vs placebo	200/40	67.5 ± 1.81	67.3 ± 2.0	1.07 ± 0.12	1.1 ± 0.14	1 year	BMD percent change, bone markers, adverse event	Low risk
Nabhan et al. (25)	RCT	Egypt	IMN(20mg daily) vs alendronate (70mg weekly)	30/30	54.7 ± 6.51	53.07 ± 6.69	0.213 ± 0.05	0.215 ± 0.05	1 year	BMD change, adverse event	Low risk
Duhan et al. (16)	RCT	India	IMN(40mg daily) vs alendronate(70mg weekly)	45/45	71 ± 5.0	71 ± 5.1	0.67 ± 0.097	0.68 ± 0.067	9 months	BMD change, adverse event	High risk

NG, nitroglycerin; ISMO, short-acting isosorbide mononitrate; ISMN, long-acting isosorbide mononitrate; IMN, isosorbide mononitrate; T, treatment; C, control; BMD percent change: (BMD at follow-up – BMD at baseline)/BMD at the baseline ×100; BMD change: BMD at follow-up – BMD at baseline.

**Table 3:** Risk of bias of randomized controlled trials evaluating the efficacy of nitrates for bone health.

Study, year	Sequence generation	Allocation concealment	Blinding of participants	Blinding of personnel	Blinding of outcome assessors	Incomplete Outcome data	Selective outcome reporting	Other sources of bias	Summary assessments of the risk of bias
Wimalawansa et al. (15)	Unclear risk	Unclear risk	Low risk	Low risk	Low risk	Low risk	Low risk	Low risk	Unclear risk
Bolland et al. (14)	Low risk	Low risk	Low risk	Low risk	Low risk	Low risk	Low risk	Low risk	Low risk
Nabhan et al. (25)	Low risk	Low risk	Low risk	Low risk	Low risk	Low risk	Low risk	Low risk	Low risk
Duhan et al. (16)	Unclear risk	Unclear risk	High risk	High risk	Low risk	Low risk	Low risk	Low risk	High risk

### Main Analysis

**Figure 2:**
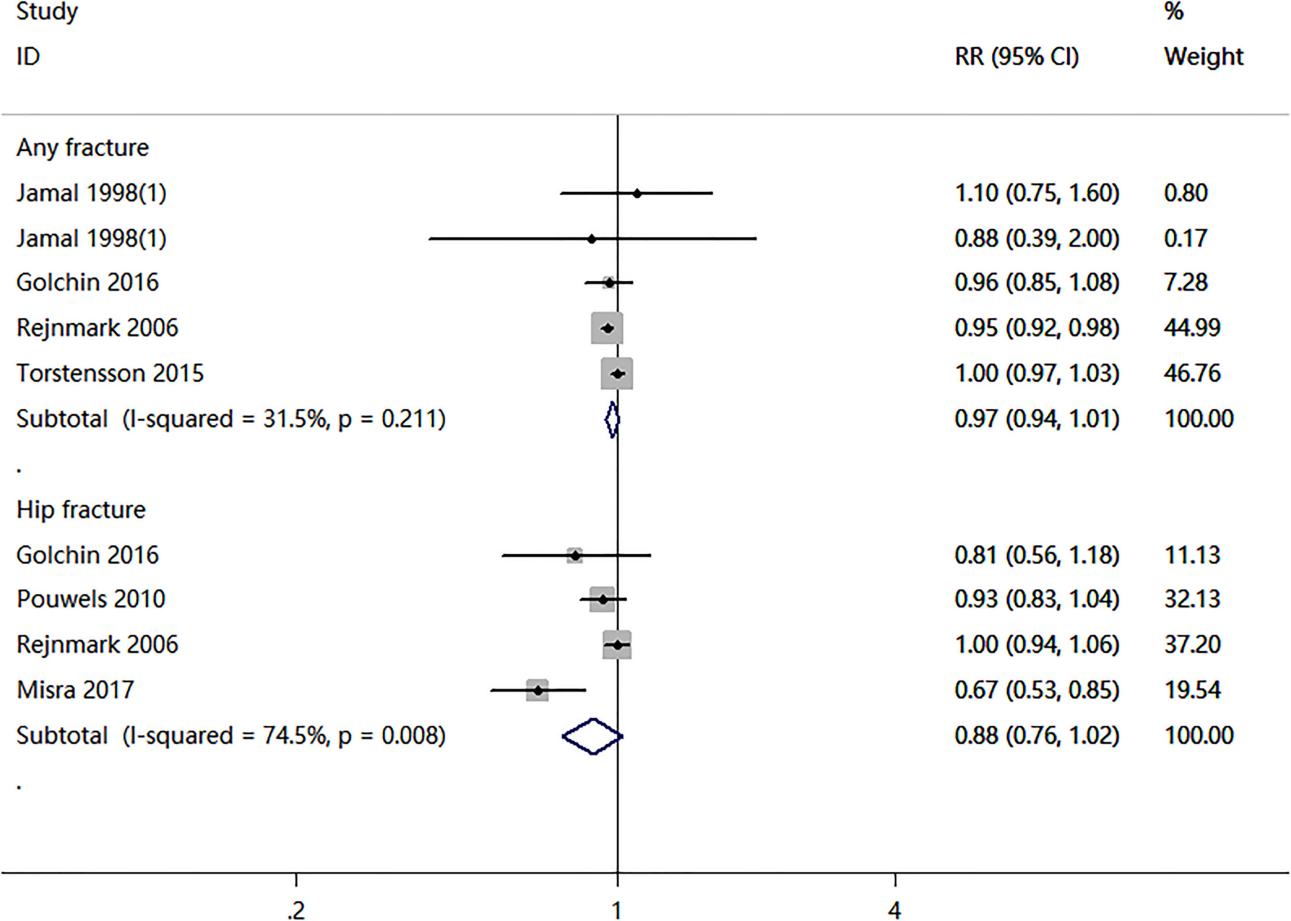
Meta-analysis results of nitrates use for the risk of any fracture and hip fracture.

**Figure 3:**
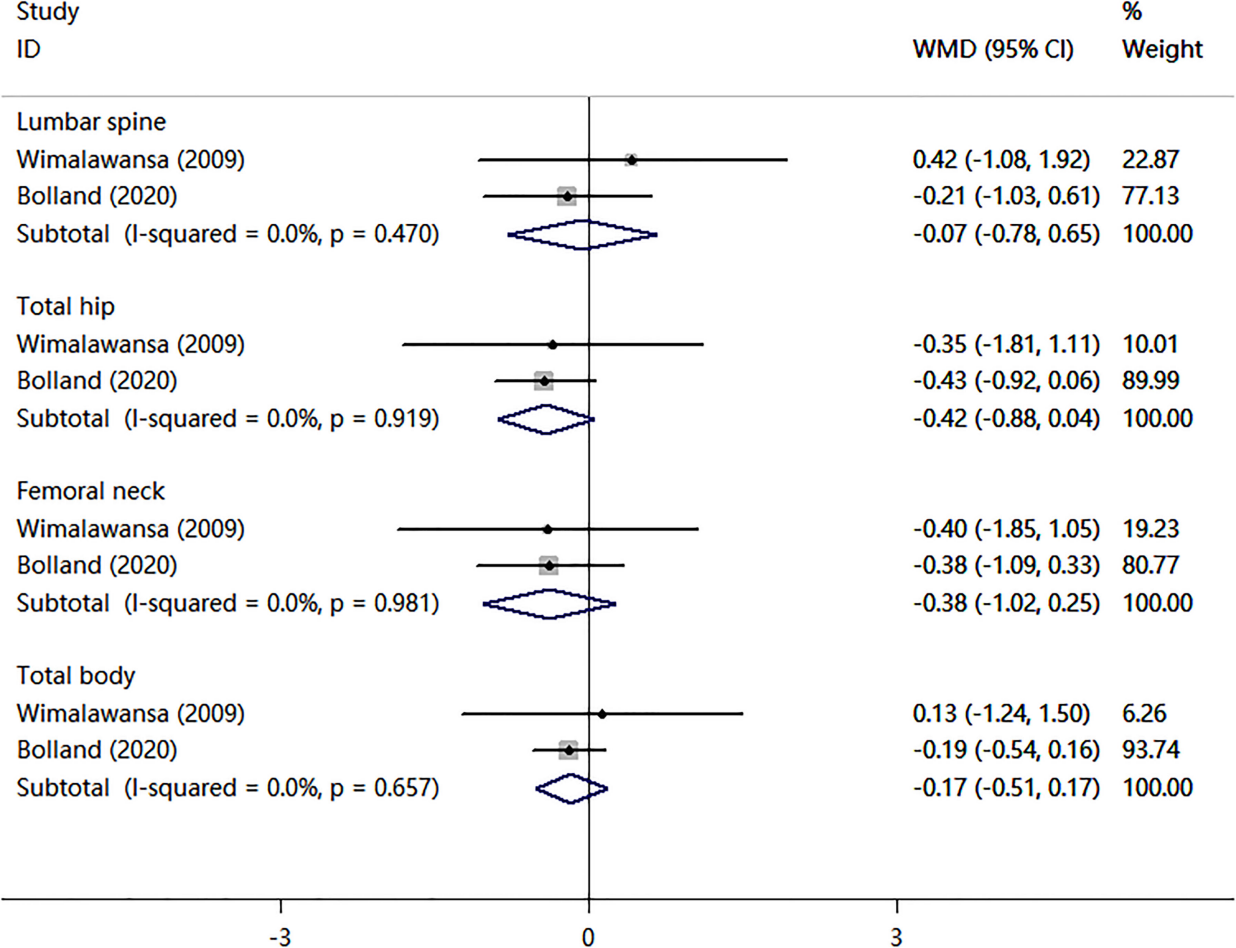
Meta-analysis of the effects of nitrates on BMD compared with placebo.

**Figure 4:**
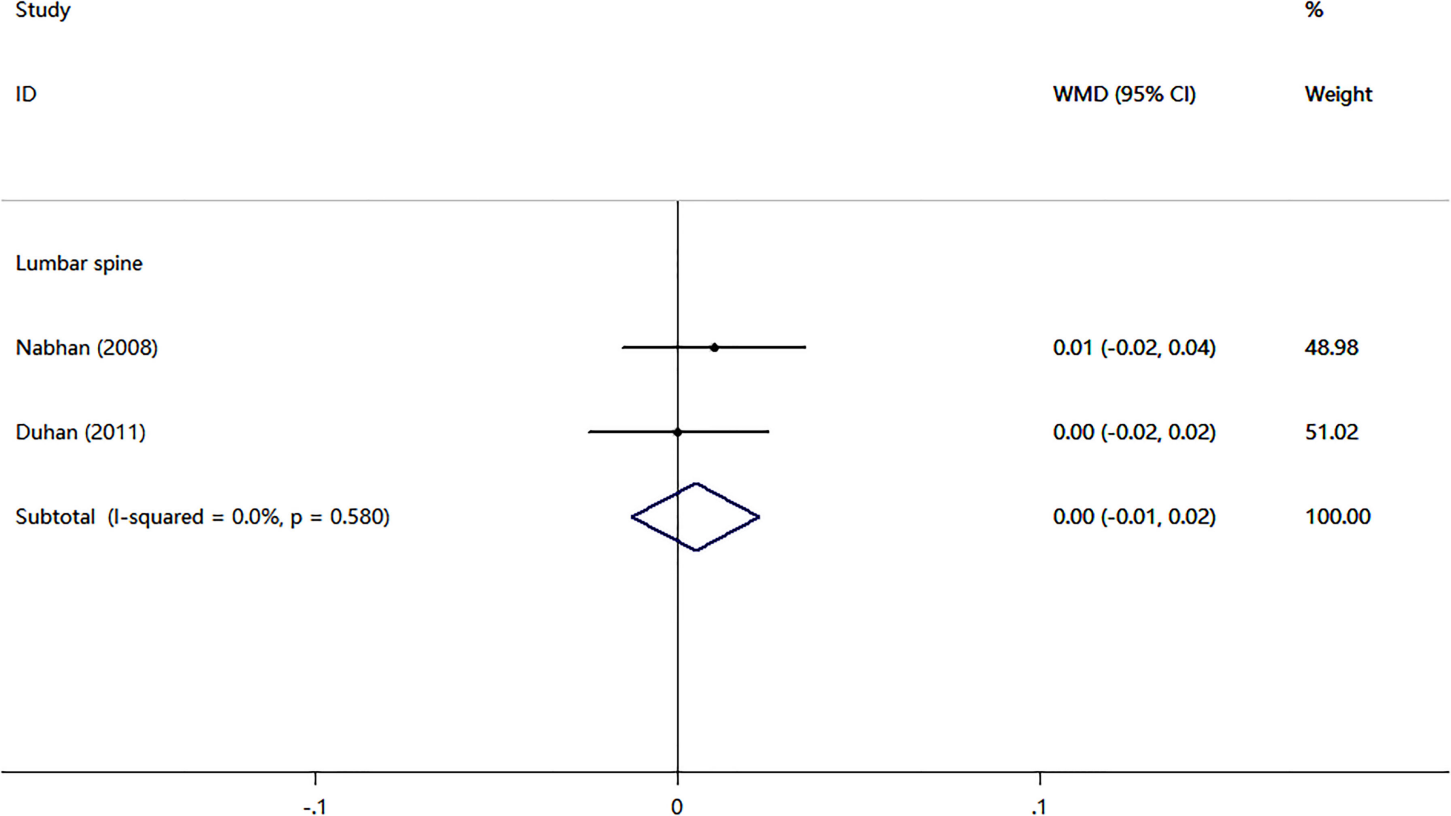
Meta-analysis of the effects of nitrates on lumbar spine BMD compared with alendronate.

Four cohort studies and two case-control studies examining the association between nitrates use and fractures were identified. As shown in **Figure 2**, the nitrates use was not associated with any fracture risk (RR = 0.97; 95% CI, 0.94–1.01; *I* ^2^ = 31.5%) and hip fracture (RR = 0.88; 95% CI, 0.76–1.02; *I* ^2^ = 74.5%). Two RCTs compared nitrates with a placebo. As shown in **Figure 3**, there were no statistically significant differences in BMD percent change at lumbar spine (WMD = -0.07, 95% CI,-0.78–0.65; *I* ^2^ = 0.0%), total hip (WMD=-0.42, 95% CI,-0.88–0.04; *I* ^2^ = 0.0%), femoral neck (WMD=-0.38, 95% CI,-1.02–0.25; *I* ^2^ = 0.0%), or total body (WMD = -0.17, 95% CI,-0.51–0.17; *I* ^2^ = 0.0%). Two randomized controlled trials (RCTs) compared nitrates with alendronate. As shown in **Figure 4**, nitrates were comparable to alendronate in increasing bone mineral density at lumbar spine (WMD = 0.00, 95% CI,-0.01–0.02; *I* ^2^ = 0.0%). Four RCTs reported on the adverse events of nitrates use (**Table 2**). The most common adverse effect was headache (14%–31.1% incidence), contributing to low adherence to therapy. Other adverse effects included palpitations, nausea, flushing, and diaphoresis.

### Subgroup Meta-Analyses

In the subgroup meta-analyses, the risk of fracture is shown in **Table 4**. When the selected studies for any fracture were grouped by study design, no significant association was seen in the three cohort studies (RR = 1.00; 95% CI, 0.97–1.03; *I* ^2^ = 0.0%). However, a negative association between the use of nitrates and any fracture risk was found only in one case-control study (RR = 0.95; 95% CI, 0.92–0.98). Two cohort and two case-control studies evaluated the association between nitrates use and hip fracture risk. The overall pooled RR for cohort studies was 0.71 (95%CI: 0.58–0.86, *I* ^2^ = 0.0%), while the pooled RR for case-control studies was 0.98 (95%CI: 0.92–1.04, *I* ^2^ = 19.3%).

**Table 4:** Subgroup analysis of nitrates use and fracture risk.

Study	No of studies	RR with 95% CI	Heterogeneity		Study	No of studies	RR with 95% CI	Heterogeneity	
			*I* ^2^(%)	*P* value				*I* ^2^(%)	*P* value
**Any fracture**					**Hip fracture**				
All	5	0.97(0.94,1.01)	31.5	0.211	All	4	0.88(0.76,1.02)	74.5	0.008
Study design					Study design				
Cohort	4	1.00(0.97,1.03)	0.0	0.858	Cohort	2	0.71(0.58,0.86)	0.0	0.399
Case control	1	0.95(0.92,0.98)	–	–	Case control	2	0.98(0.92,1.04)	19.3	0.266
NOS score					NOS score				
9 point	2	0.97(0.93,1.03)	81.2	0.021	9 point	2	0.98(0.92,1.04)	19.3	0.266
8 point	3	0.97(0.87,1.09)	0.0	0.776	8 point	2	0.71(0.58,0.86)	0.0	0.399
Region					Region				
North America	3	0.97(0.87,1.09)	0.0	0.776	North America	1	0.81(0.56,1.18)	–	–
Europe	2	0.97(0.93,1.03)	81.2	0.021	Europe	3	0.89(0.76,1.05)	81.7%	0.004

Grouping of studies by NOS score revealed no significant association between the nitrates use and the any fracture risk in both the 9 point groups (RR = 0.97; 95% CI, 0.93–1.03; *I* ^2^ = 81.2%) and 8 point groups (RR =0.97; 95% CI, 0.87–1.09; *I* ^2^ = 0.0%). However, there was a significant association of nitrates with hip fracture in 8 point groups (RR, 0.71; 95% CI, 0.58–0.89; *I* ^2^ = 0.0%), but no significant association in 9 point groups (RR, 0.98; 95% CI, 0.92–1.04; *I* ^2^ = 19.3%)

When we grouped studies by region, we found no significant association between the nitrates use and the any fracture risk in North America (RR = 0.97; 95% CI, 0.87–1.09; *I* ^2^ = 0.0%) and Europe (RR = 0.97; 95% CI, 0.93–1.03; *I* ^2^ = 81.2%). The pooled RR for the hip fracture risk of North American people with nitrates was 0.81 (95%CI: 0.56–1.18), and the pooled RR for the hip fracture risk of European people with nitrates was 0.89 (95%CI: 0.76–1.05, *I* ^2^ = 81.7%).

### Sensitivity Analysis and Publication Bias

The results of the sensitivity analysis demonstrated the stability of outcomes in meta-analyses(**Supplementary Figures 1, 2**). No indication of publication bias was found for studies that reported any fracture risk (Begg *P* = 1.000; Egger *P* = 0.983) and hip fracture (Begg *P* = 0.139; Egger *P* = 0.308) (**Supplementary Figures 3, 4**).

## Discussion

In this meta-analysis of 10 studies, we found that nitrates use was not associated with a reduced risk of any fracture or hip fracture in observational studies. The results of four randomized controlled trials on the effects of nitrates on BMD were inconsistent. There were no statistically significant differences in BMD percent change at any sites in these two RCTs compared with a placebo (14, 15). In contrast, nitrates and alendronate had similar effects in increasing bone BMD in another two RCTs (16, 25).

NO is a short-lived free radical that regulates a variety of physiological processes, including bone remodeling (26). In the acid environment of the stomach, NO can be created nonenzymatically from nitrites. Organic nitrates (nitroglycerin, isosorbide mononitrate, isosorbide dinitrate) can operate as NO donors (27). Intermediate dosages of NO have been demonstrated to improve skeletal health in several studies. However, the benefits of NO supplements on bone mass have been controversial. Numerous *in vivo* animal studies have demonstrated that NO donors help to decrease bone resorption while also improving bone growth (10, 28, 29). NO appears to have a biphasic effect on bone-forming cells, promoting bone growth at low doses while inhibiting bone formation at higher concentrations (30). Because nitroglycerin has a somewhat narrow therapeutic window for osteoporosis treatment, the proper dose must be employed to get positive BMD results (15). Continuous exposure to nitrates may promote tachyphylaxis in bone, just as it does with angina symptom management. Once-daily treatment of nitroglycerin ointment enhanced BMD in ovariectimized rats, but more frequent application had little effect (31). Based on this potential for tachyphylaxis, randomized controlled trials using once-daily dosing of nitroglycerin ointment would not achieve satisfactory results for bone health. The most well-known study on nitrates found that nitroglycerin improved BMD by 6% to 7% at all sites over 24 months, with significant increases in markers of bone formation and decreases in markers of bone resorption, but the study was retracted five years later (32). Another observational study (33) reported that nitrate use was associated with increased BMD at the hip and spine in men and women. It was also retracted. Two articles about the results of nitrates and alendronate have similar effects in increasing bone BMD and should be carefully considered. More randomized control trials are needed to determine the effects of nitrates on bone health.

Our meta-analysis has several strengths. This meta-review was the first to review the efficacy of nitrates for bone health. In addition, it examined the associations stratified by the type of fracture, the study design, NOS score, and region. However, our meta-analysis has some limitations as well. First, due to the small number of RCT studies, the results of our meta-analysis of RCTs are highly heterogeneous. Second, we may have missed unpublished studies and those that were not in English, resulting in an overestimation of the efficacy of these treatments. Third, we were unable to conduct a meta-analysis on adverse events, because many studies failed to report different adverse events.

## Conclusion

This meta-analysis of observational data found no association between nitrate use and fracture risk. The results of RCTs on the usage of nitrates and their effects on BMD are contradictory. Further well-designed trials confirming their benefit for bone health are required before it can be recommended for routine use.

## Data Availability Statement

The original contributions presented in the study are included in the article/**Supplementary Material**. Further inquiries can be directed to the corresponding author.

## Author Contributions

WL and GW designed the study and collected the data. WL drafted the manuscript. ZM contributed to the writing. GW provided critical feedback and contributed to the review of the manuscript. All authors contributed to the article and approved the submitted version.

## Conflict of Interest

The authors declare that the research was conducted in the absence of any commercial or financial relationships that could be construed as a potential conflict of interest.

## Publisher’s Note

All claims expressed in this article are solely those of the authors and do not necessarily represent those of their affiliated organizations, or those of the publisher, the editors and the reviewers. Any product that may be evaluated in this article, or claim that may be made by its manufacturer, is not guaranteed or endorsed by the publisher.
