# Supplementary material for: The Efficacy of Nitrates for Bone Health: A Systematic Review and Meta-Analysis of Observational and Randomized Controlled Studies
Source: Front Endocrinol (Lausanne). 2022 Feb 10;13:833932. doi: 10.3389/fendo.2022.833932 (PMC8867074; doi:10.3389/fendo.2022.833932)

**Supplementary Table 1**. Summary of search strategies.

| **Pubmed** | | |
| --- | --- | --- |
| Search | Query | Results |
| #1 | ((((((((((((nitrate[Title/Abstract]) OR (nitrates[Title/Abstract])) OR (nitroglycerin[Title/Abstract])) OR (glyceryl trinitrate[Title/Abstract])) OR (nitric oxide donors[Title/Abstract])) OR (isosorbide dinitrate[Title/Abstract])) OR (isosorbide[Title/Abstract])) OR (dinitrate[Title/Abstract])) OR (mononitrogen[Title/Abstract])) OR (vasodilator agents[Title/Abstract])) OR (organic nitrates[Title/Abstract])) OR (isosorbide mononitrate[Title/Abstract])) OR (sodium nitroprusside[Title/Abstract]) | 97284 |
| #2 | ("Osteoporosis"[Mesh]) OR ((((((((((((((((((((Osteoporoses[Title/Abstract]) OR (Osteoporosis, Post-Traumatic[Title/Abstract])) OR (Osteoporosis, Post Traumatic[Title/Abstract])) OR (Post-Traumatic Osteoporoses[Title/Abstract])) OR (Post-Traumatic Osteoporosis[Title/Abstract])) OR (Osteoporosis, Senile[Title/Abstract])) OR (Osteoporoses, Senile[Title/Abstract])) OR (Senile Osteoporoses[Title/Abstract])) OR (Osteoporosis, Involutional[Title/Abstract])) OR (Senile Osteoporosis[Title/Abstract])) OR (Osteoporosis, Age-Related[Title/Abstract])) OR (Osteoporosis, Age Related[Title/Abstract])) OR (Bone Loss, Age-Related[Title/Abstract])) OR (Age-Related Bone Loss[Title/Abstract])) OR (Age-Related Bone Losses[Title/Abstract])) OR (Bone Loss, Age Related[Title/Abstract])) OR (Bone Losses, Age-Related[Title/Abstract])) OR (Age-Related Osteoporosis[Title/Abstract])) OR (Age-Related Osteoporoses[Title/Abstract])) OR (Osteoporoses, Age-Related[Title/Abstract])) | 60658 |
| #3 | ("Bone Density"[Mesh]) OR (((((((Bone Densities[Title/Abstract]) OR (Density, Bone[Title/Abstract])) OR (Bone Mineral Density[Title/Abstract])) OR (Bone Mineral Density[Title/Abstract])) OR (Density, Bone Mineral[Title/Abstract])) OR (Bone Mineral Content[Title/Abstract])) OR (Bone Mineral Contents[Title/Abstract])) | 72363 |
| #4 | ("Fractures, Bone"[Mesh]) OR (((((((((((((((Broken Bones[Title/Abstract]) OR (Bone, Broken[Title/Abstract])) OR (Bones, Broken[Title/Abstract])) OR (Broken Bone[Title/Abstract])) OR (Bone Fractures[Title/Abstract])) OR (Bone Fracture[Title/Abstract])) OR (Fracture, Bone[Title/Abstract])) OR (Spiral Fractures[Title/Abstract])) OR (Fracture, Spiral[Title/Abstract])) OR (Fractures, Spiral[Title/Abstract])) OR (Spiral Fracture[Title/Abstract])) OR (Torsion Fractures[Title/Abstract])) OR (Fracture, Torsion[Title/Abstract])) OR (Fractures, Torsion[Title/Abstract])) OR (Torsion Fracture[Title/Abstract])) | 202272 |
| #5 | #2 OR#3 OR #4 | 286091 |
| #6 | #1 AND #5 | 133 |
| **Embase** | | |
| #1 | 'nitrate'/exp OR nitrates:ab,ti OR nitroglycerin:ab,ti OR 'glyceryl trinitrate':ab,ti OR 'nitric oxide donors':ab,ti OR 'isosorbide dinitrate':ab,ti OR isosorbide:ab,ti OR dinitrate:ab,ti OR mononitrogen:ab,ti OR 'vasodilator agents':ab,ti OR 'organic nitrates':ab,ti OR 'isosorbide mononitrate':ab,ti OR 'sodium nitroprusside':ab,ti | 102256 |
| #2 | 'osteoporosis'/exp OR 'post-traumatic osteoporoses':ab,ti OR osteoporoses:ab,ti OR 'osteoporosis, post-traumatic':ab,ti OR 'osteoporosis, post traumatic':ab,ti OR 'post-traumatic osteoporosis':ab,ti OR 'osteop(1)orosis, senile':ab,ti OR 'osteoporoses, senile':ab,ti OR 'senile osteoporoses':ab,ti OR 'osteoporosis, involutional':ab,ti OR 'senile osteoporosis':ab,ti OR 'osteoporosis, age-related':ab,ti OR 'osteoporosis, age related':ab,ti OR 'bone loss, age-related':ab,ti OR 'age-related bone loss':ab,ti OR 'age-related bone losses':ab,ti OR 'bone loss, age related':ab,ti OR 'bone losses, age-related':ab,ti OR 'age-related osteoporosis':ab,ti OR 'age-related osteoporoses':ab,ti OR 'osteoporoses, age-related':ab,ti OR 'tiosteopenic postmenopausal women':ab,ti OR 'low bone mass':ab,ti OR 'low bone mineral density':ab,ti OR osteopenia:ab,ti OR 'postmenopausal women':ab,ti | 192599 |
| #3 | 'bone density'/exp OR 'bone densities':ab,ti OR 'density, bone':ab,ti OR 'bone mineral density':ab,ti OR 'density, bone mineral':ab,ti OR 'bone mineral content':ab,ti OR 'bone mineral contents':ab,ti | 112884 |
| 4 | 'fracture'/exp OR 'broken bones':ab,ti OR 'bone, broken':ab,ti OR 'bones, broken':ab,ti OR 'broken bone':ab,ti OR 'bone fractures':ab,ti OR 'bone fracture':ab,ti OR 'fracture, bone':ab,ti OR 'spiral fractures':ab,ti OR 'fracture, spiral':ab,ti OR 'fractures, spiral':ab,ti OR 'spiral fracture':ab,ti OR 'torsion fractures':ab,ti OR 'fracture, torsion':ab,ti OR 'fractures, torsion':ab,ti OR 'torsion fracture':ab,ti | 358586 |
| #5 | #2 OR#3 OR #4 | 541789 |
| #6 | #1 AND #5 | 531 |
| #7 | #4 AND #10 AND [humans]/lim AND [clinical study]/lim | 286 |
| **Cochrane library** | | |
| #1 | MeSH descriptor: [Nitrates] explode all trees | 1058 |
| #2 | nitrates:ti,ab,kw OR nitroglycerin:ti,ab,kw OR glyceryl trinitrate:ti,ab,kw OR nitric oxide donors:ti,ab,kw OR isosorbide dinitrate:ti,ab,kw OR isosorbide:ti,ab,kw OR dinitrate:ti,ab,kw OR mononitrogen:ti,ab,kw OR vasodilator agents:ti,ab,kw OR organic nitrates:ti,ab,kw OR isosorbide mononitrate:ti,ab,kw OR sodium nitroprusside:ti,ab,kw | 11381 |
| #3 | MeSH descriptor: [Osteoporosis] explode all trees | 4287 |
| #4 | Osteoporoses:ti,ab,kw OR Osteoporosis, Post-Traumatic:ti,ab,kw OR Osteoporosis, Post Traumatic:ti,ab,kw OR Post-Traumatic Osteoporoses:ti,ab,kw OR Post-Traumatic Osteoporosis:ti,ab,kw | 5941 |
| #5 | MeSH descriptor: [Bone Density] explode all trees | 4815 |
| #6 | bone densities:ti,ab,kw OR density, bone:ti,ab,kw OR bone mineral density:ti,ab,kw OR density, bone mineral:ti,ab,kw OR bone mineral content:ti,ab,kw OR bone mineral contents:ti,ab,kw | 13594 |
| #7 | fracture:ti,ab,kw OR fractures:ti,ab,kw OR broken bones:ti,ab,kw OR bone, broken:ti,ab,kw OR bones, broken:ti,ab,kw OR broken bone:ti,ab,kw OR bone fractures:ti,ab,kw OR bone fracture:ti,ab,kw OR fracture, bone:ti,ab,kw OR spiral fractures:ti,ab,kw OR fracture, spiral:ti,ab,kw OR fractures, spiral:ti,ab,kw OR spiral fracture:ti,ab,kw OR torsion fractures:ti,ab,kw OR fracture, torsion:ti,ab,kw OR fractures, torsion:ti,ab,kw OR torsion fracture:ti,ab,kw | 24808 |
| #8 | #1 OR #2 | 11534 |
| #9 | #3 OR #4 | 10225 |
| #10 | #5 OR #6 | 13625 |
| #11 | #9 OR #10 OR #7 | 40520 |
| #12 | #8 AND #11 | 52 |


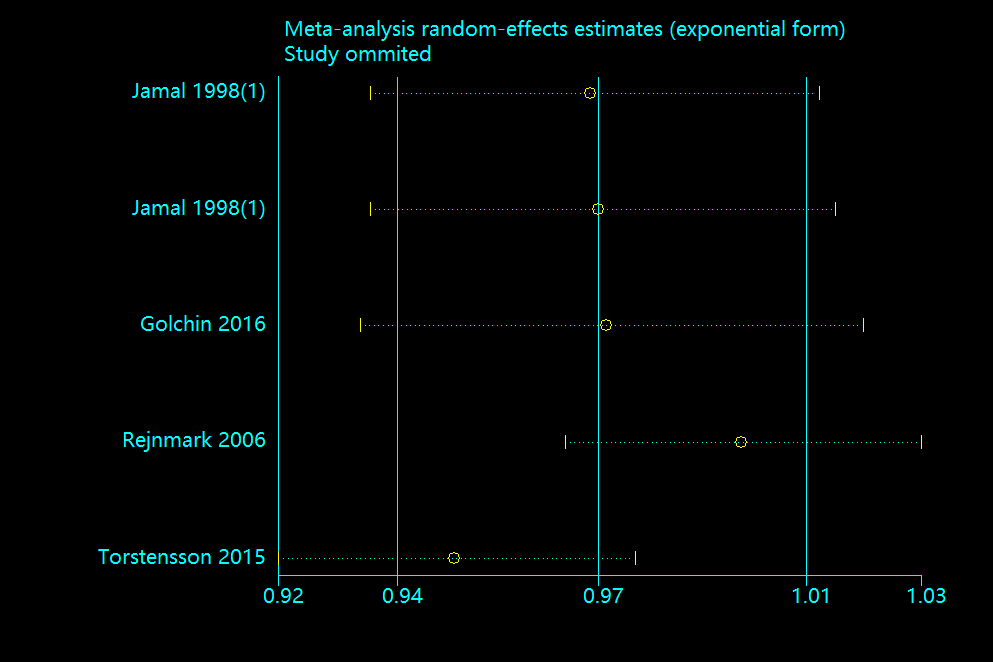


Supplementary Figure 1. The results of the sensitivity analysis for studies that reported any fracture risk.


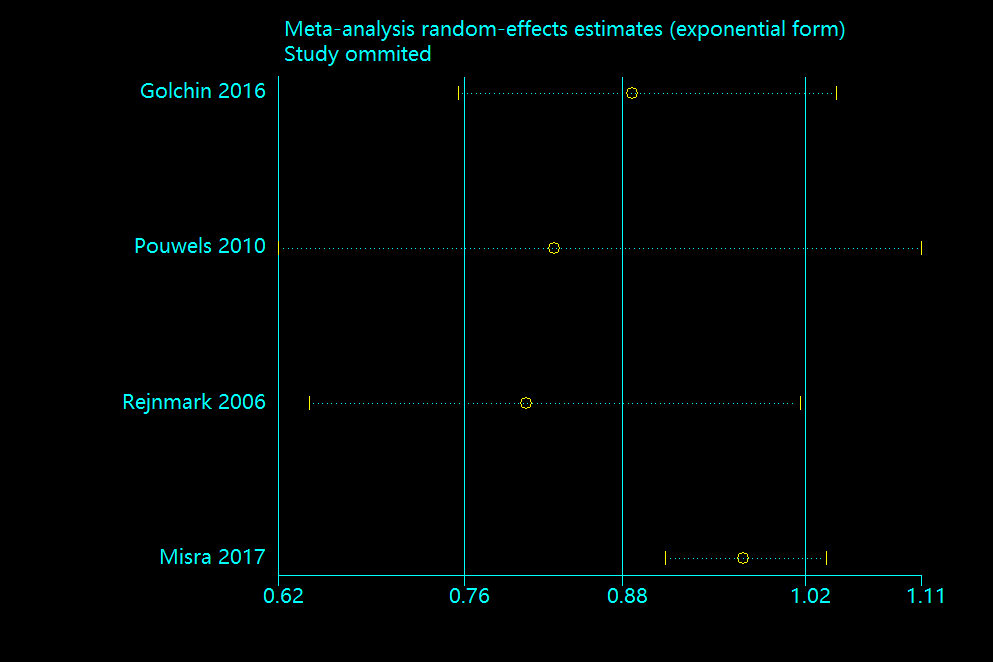


Supplementary Figure 2. The results of the sensitivity analysis for studies that reported hip fracture risk.

Supplementary Figure 3. The results of the Begg’s and Egger’s test for studies that reported any fracture risk.


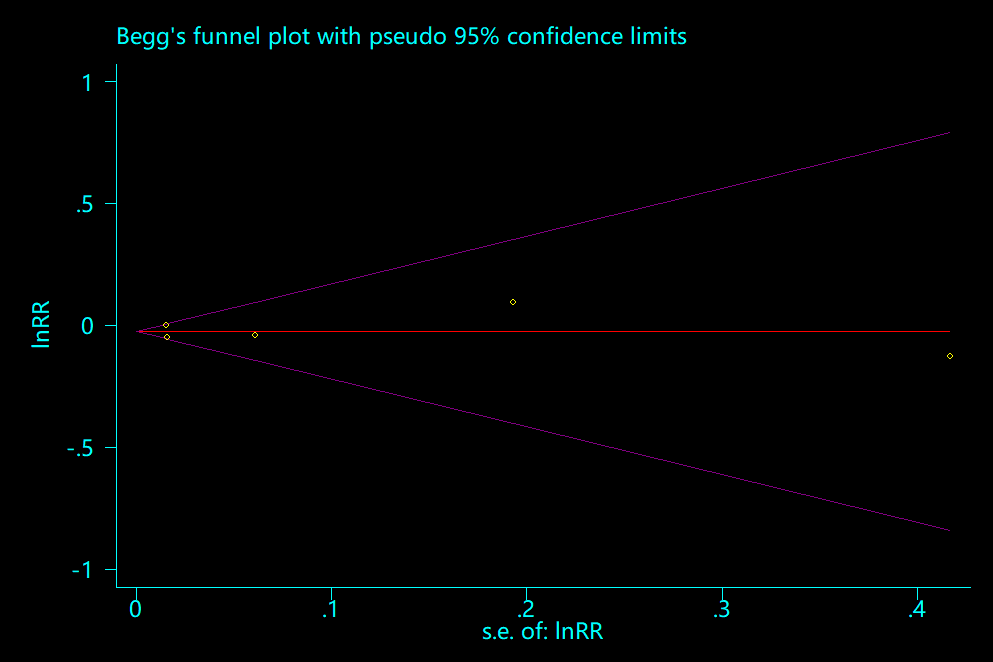

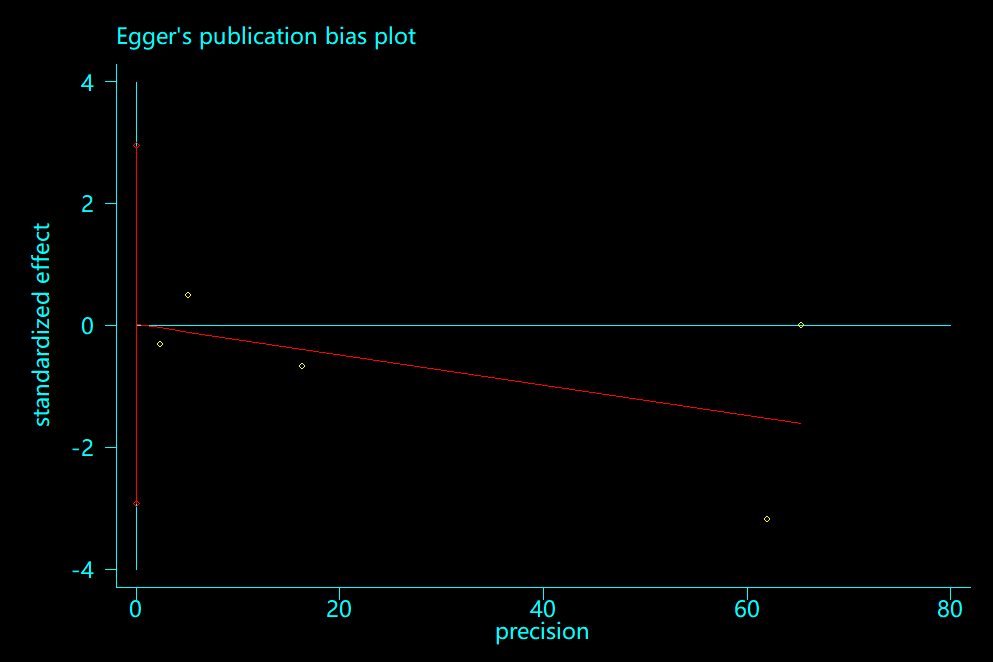


Supplementary Figure 4. The results of the Begg’s and Egger’s test for studies that reported hip fracture risk.


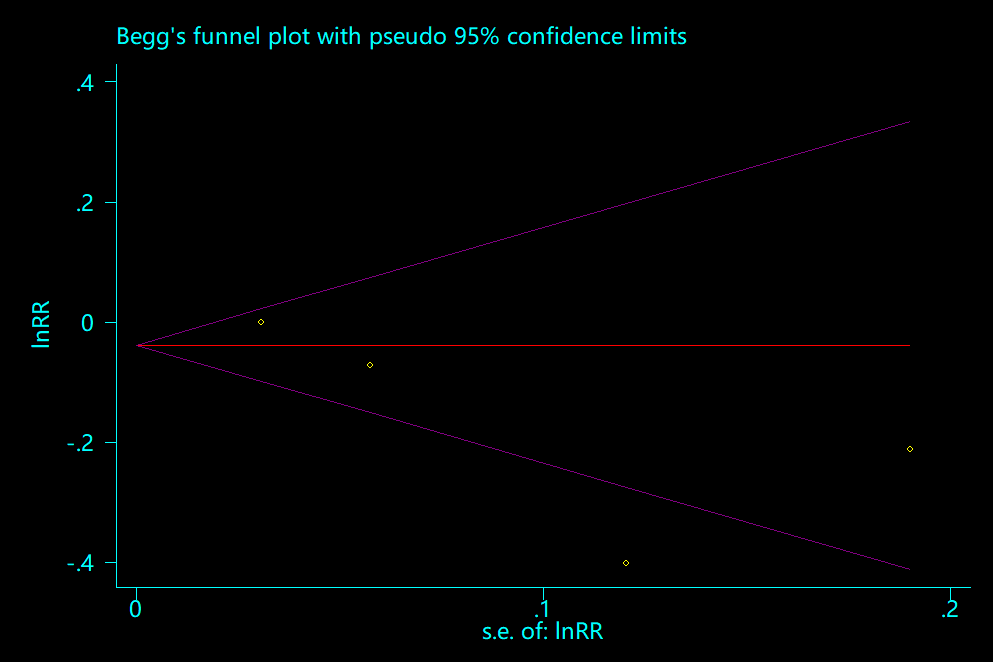

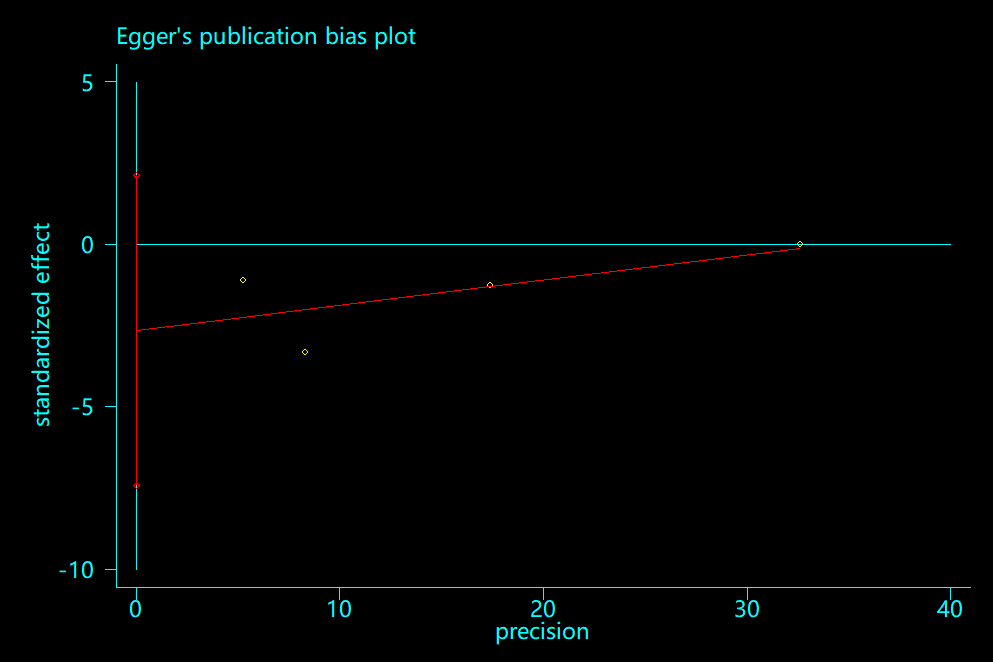

Supplement: Supplementary file 1 [file DataSheet_1.docx]
